# The Dark Side of Energy Drinks: A Comprehensive Review of Their Impact on the Human Body

**DOI:** 10.3390/nu15183922

**Published:** 2023-09-09

**Authors:** Andrea Costantino, Aniello Maiese, Julia Lazzari, Chiara Casula, Emanuela Turillazzi, Paola Frati, Vittorio Fineschi

**Affiliations:** 1 Institute of Legal Medicine, Department of Surgical, Medical and Molecular Pathology and Critical Care Medicine, University of Pisa, Via Roma, 55, 56126 Pisa, Italy; a.costantino8@studenti.unipi.it (A.C.); aniello.maiese@unipi.it (A.M.); lazzari.jul@gmail.com (J.L.); c.casula5@studenti.unipi.it (C.C.); emanuela.turillazzi@unipi.it (E.T.); 2 Institute of Legal Medicine, Department of Anatomical, Histological, Forensic and Orthopedic Sciences, Sapienza University of Rome, Viale Regina Elena 336, 00161 Rome, Italy; paola.frati@uniroma1.it

**Keywords:** energy drink, Red Bull, Monster, taurine, adverse effects, arrhythmia, death

## Abstract

In recent years, the consumption of energy drinks by young adults and athletes has risen significantly, but concerns have been raised about the potential health risks associated with excessive consumption. These concerns include cardiovascular problems, nervous system disorders, and the potential for addiction. This review aims to examine the reported effects of acute or chronic abuse of energy drinks on human health. The analysis shows a significant prevalence of adverse effects, particularly on the cardiovascular and neurovegetative systems. In particular, the analysis identified nine cases of cardiac arrest, three of which were fatal. The aetiology of these adverse effects is attributed to the inherent neurostimulant properties of these beverages, of which caffeine is the predominant component. A comparison of documented effects in humans with experimental studies in animal models showed an overlap in results. This review highlights the need for greater rigour in the assessment of sudden cardiac death, particularly in young people, as legal substances such as energy drinks may be involved. We propose stricter limits on the consumption of these beverages than for caffeine, based on the evidence found and the data in the literature. This review also calls for the establishment of regulations governing the consumption of these products in view of their potential impact on human health.

## 1. Introduction

The Food and Drug Administration (FDA) defines energy drinks (EDs) as “a class of products in liquid form that typically contains caffeine, with or without other added ingredients.” They typically contain large amounts of caffeine, added sugars, other additives, and legal stimulants such as guarana, taurine, and L-carnitine. These legal stimulants can increase alertness, attention, and energy, as well as increasing blood pressure, heart rate, and breathing. These products are marketed as enhancers of mental acuity and physical performance [1,2]. Prominent examples of energy drinks include Red Bull, Monster, NOS, Rockstar, Lucozade, Eastroc Super Drink, Bang Energy, and 5 Hour Energy [3,4], as described in Table 1. Adolescents gravitate towards these beverages to swiftly boost energy levels, enhance alertness, and increase scholastic or athletic performance.

Consequences of this consumption pattern have led to a rising incidence of young individuals seeking medical attention in emergency departments due to an array of adverse health outcomes, as documented in results section. Reports underscore that energy drinks have deleterious effects on a broad spectrum of bodily organs, culminating in mild adversities such as anxiety, gastrointestinal disturbances, dehydration, nervousness, and tachycardia, along with more severe outcomes like rhabdomyolysis, acute kidney injury (AKI), ventricular fibrillation, seizures, acute mania, and stroke. Furthermore, instances linking energy drink consumption to fatalities have been documented.

The rise of the energy drink market, particularly within the younger demographic, has caused a 70% escalation in caffeine ingestion among caffeine-consuming children and adolescents from 1977 to 2009 [1]. Data sourced from the National Health and Nutrition Examination Survey spotlight an average caffeine intake of 61 mg daily for teenagers [5]. While youth caffeine consumption has receded in recent decades, the utilisation of energy drinks has concurrently surged [5].

These beverages substantially differ in both caffeine content (ranging from 50 to 505 mg per can or bottle) and caffeine concentration (ranging from 2.5 to 171 mg per 28 mL). By comparison, a 170 g cup of brewed coffee contains caffeine concentrations varying between 77 and 150 mg [3].

As shown in Table 1, energy drinks are composed of a variety of ingredients, including taurine, ginseng, sugar, guarana, etc.

**Table 1:** Most popular energy drinks and ingredients per 500 mL [3,4,6].

Energy Drink	Caffeine (mg)	Sugar (g)	Other Ingredients
Red Bull	160	54	Taurine (2000 mg), gluconolactone (1200 mg), inositol N/S [not specified] and vitamins B3, B5, B6 and B12 N/S
Monster	160	54	Taurine (2000 mg), gluconolactone N/S, carnitine N/S, inositol and guarana N/S, ginseng (400 mg) and vitamins B2, B3, B6 and B12 (40 mg)
Rockstar	160	62	Taurine (2000 mg), carnitine (50 mg), inositol (50 mg) and guarana (50 mg), ginseng (50 mg), gingko biloba (300 mg), milk thistle (40 mg) and vitamins B2, B3, B5, B6 and B12 (50 mg)
Mountain Dew	72	61	Carbonated water, high-fructose corn syrup, concentrated orange juice, citric acid, natural flavour, sodium benzoate, caffeine, sodium citrate, gum arabic, erythorbic acid, calcium disodium, yellow 5
Race	160	0	N/S
Sting	290	50	Taurine (148 mg), inositol (21 mg), ginseng (9.7 mg), vitamin B3 (10.2 mg)
Magnus Omnilife	N/S	N/S	N/S
Demon Energy Shot (sold in 60 mL)	1600 (200 mg per 60 mL)	N/S	Taurine (10 g), glucuronolactone (N/S), inositol (N/S), B3 (N/S), guarana (60 mg), B5 (N/S), B6 (N/S), B12 (N/S)
Full Throttle	141	57	Taurine (N/S), guarana (N/S), B3 (N/S), B6 (N/S), B8 (N/S), carnitine (N/S)
Lucozade	60.5	23	Carbonated water (N/S), citric acid (N/S), sodium gluconate (N/S), potassium sorbate (N/S), aspartame (N/S), acesulfame K (N/S), flavourings (N/S), sunset yellow (N/S), ponceau 4R (N/S), ascorbic acid (N/S).

In particular, taurine and gluconolactone are claimed to be the main components responsible for the effects attributed to Red Bull. Taurine, a derivative of the amino acid cysteine, is found abundantly in cardiac and skeletal muscles [7,8]. Its engagement covers various physiological functions encompassing neuromodulation, cell membrane stabilisation, and the regulation of intracellular calcium levels [9]. Acknowledged for its anti-arrhythmic attributes, taurine’s capacity for cation transport regulation contributes to its effect [10]. It is instrumental in modulating the inwardly rectifying K+ current and action potential duration in cardiac muscles [11], along with inhibiting the fast Na+ current, thereby evoking class I antiarrhythmic activity [12]. Its presence in significant concentrations within the brain underscores its pivotal role in neuroprotection and neurotransmission enhancement [13]. The prospect of taurine in tandem with caffeine bolstering concentration, reaction time, and emotional state has sparked investigation, although conclusive evidence on combinatorial cognitive effects remains elusive. Seidl et al. conducted a double-blind, placebo-controlled trial administering caffeine, taurine, and glucuronolactone to the experimental group, yielding shorter motor reaction times and higher emotional well-being scores [14]. While the study implied a positive cognitive impact, GABAergic, glycinergic, cholinergic, and adrenergic neurotransmitter system interactions were posited, acknowledging the caffeine factor [15].

Gluconolactone, an outcome of hepatic glucose metabolism, stands as a precursor for ascorbic acid synthesis. In the 1960s, Japanese researchers [16] directed attention toward its performance-amplifying attributes. A study demonstrated enhanced swimming endurance in laboratory rats following the direct intestinal injection of glucuronolactone, glucose, glycogen, and other agents, with the former group outperforming in two of three instances. The findings suggest that the equivalent human dose could range from 1 to 2 g of glucuronolactone, compared to 600 mg in a Red Bull can. Detoxifying potential may contribute to these results, as glucuronolactone supplementation may fortify the body’s natural defences against carcinogens and tumour promoters [8].

Among the additional ingredients commonly found within energy drinks, carnitine, guarana, and the vitamin B complex should be mentioned.

Carnitine, comprising several compounds, including L-carnitine, acetyl-L-carnitine, and propionyl-L-carnitine [17], emerges as a derivative of an amino acid. It occurs naturally in numerous foods, particularly animal-derived foods, and is available in dietary supplement form. Carnitine synthesis transpires endogenously within the liver, kidneys, and brain from the amino acids, lysine and methionine [18,19]. This compound plays a pivotal role in energy production, serving as an indispensable cofactor that facilitates the transport of long-chain fatty acids into mitochondria for oxidation, leading to adenosine triphosphate (ATP) energy generation [20,21].

Guarana (*Paullinia cupana*), a climbing plant native to the Amazon, has historically served as a stimulant and traditional medicine among Brazil’s indigenous peoples [22]. Guarana seeds notably surpass coffee beans in caffeine content, containing additional xanthine alkaloids such as theobromine and theophylline [23]. This botanical additive enhances the caffeine content and stimulatory attributes of energy drinks (EDs), with its caffeine content being unlisted on product labels due to its status as an herbal supplement [24].

Comprising eight B vitamins, the vitamin B complex includes thiamine (B1), riboflavin (B2), niacin (B3), pantothenic acid (B5), pyridoxine hydrochloride (B6), biotin (B7), inositol (B8), and cyanocobalamin (B12). These vitamins act as coenzymes that are integral to proper cellular function, particularly in mitochondrial activity and energy production. Hence, there is some conjecture that B vitamins might increase energy expenditure [25].

The aim of this review is to summarise all evidence on the adverse effects of energy drink consumption.

## 2. Materials and Methods

**Figure 1:**
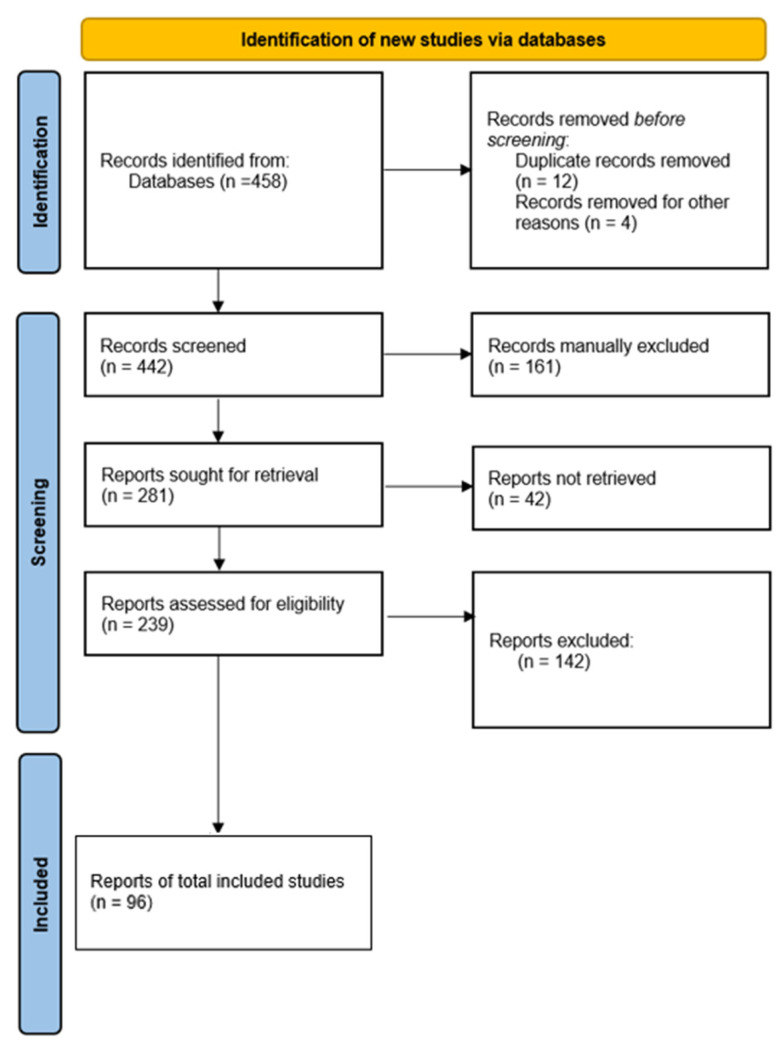
Our review strategy following PRISMA standards.

This systematic review follows the Preferred Reporting Items for Systematic Review (PRISMA) standards [26] (Figure 1). In the context of specific events, such as acute intoxication or preliminary reports of legal substances, we believe that case reports/studies and case series involving human subjects with medical reports can provide valuable evidence for systematic reviews. Therefore, descriptive observational study designs, including case series, individual case reports, and descriptive cross-sectional studies, were considered for inclusion in this review. We conducted a comprehensive literature search and critically appraised the collected studies. An electronic search was conducted using PubMed, Google Scholar and EBSCO search engines to identify peer-reviewed articles published between 5 January 2009 and 30 April 2023, using the search terms ‘energy drink’, ‘Red Bull’, ‘Monster’, ‘taurine’, ‘adverse effects’, ‘arrhythmia’, ‘renal failure’, ‘death’, ‘gastrointestinal’ in the title, abstract and keywords. Internet search engines such as Google were also used to find relevant information. In addition, the reference lists of all retrieved papers were reviewed and cross-referenced to identify additional relevant literature. Only English-language papers were included in this study. Data on each case were extracted, including age and sex of the case, brand of energy drink consumed (some brands were unknown), main pathologies, type of event and onset. Two or more independent reviewers screened titles and abstracts against the inclusion criteria for the review. The results of the search and study inclusion process were reported in detail in the final systematic review according to the Preferred Reporting Items for Systematic Reviews and Meta-analyses (PRISMA) guidelines. In addition, case–control studies using animal models were included in the review to compare data from human case reports. The above search identified 458 articles, which were screened to exclude duplicates. The resulting reference lists were then screened for titles and abstracts, leaving 442 articles for further consideration. Non-English articles were excluded. The inclusion criteria were as follows: (1) original research articles and (2) case reports/series. These publications were carefully assessed, taking into account the main objectives of the review. Reviews and mini-reviews were not included in the qualitative synthesis but were used to check for missing articles. After this evaluation, 96 scientific papers remained.

## 3. Results

The papers in our study were divided in seven groups: cardiac effects (35 papers), gastrointestinal effects (12 papers), neurologic effects (18 papers), renal effects (7), gynaecological effects (2 papers), autoimmune and skin effects (2 papers). Table 1, Table 2, Table 3, Table 4, Table 5, Table 6 and Table 7 show brief descriptions of these seven groups of studies, respectively. Furthermore, we incorporated case–control studies utilizing animal models (20 papers).

**Figure 2:**
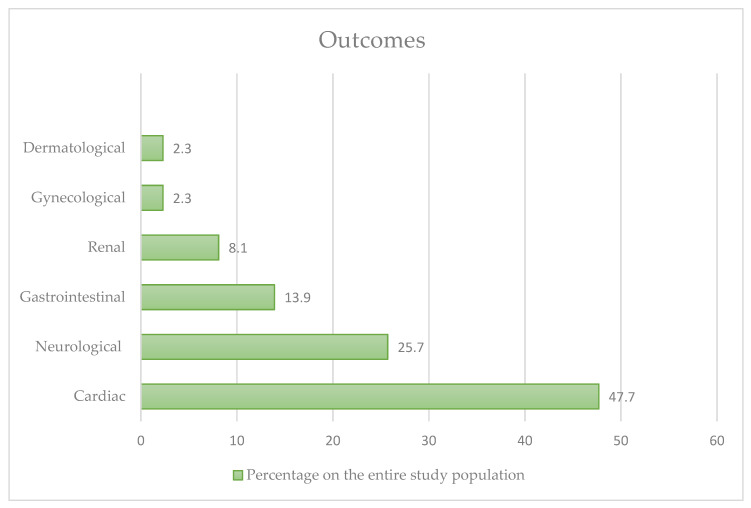
Different outcomes in our review.

We evaluated a total of 86 cases (Figure 2). Most of the patients were young (median age, 30 years; range, 8 to 62 years). Slightly more men (66 patients, 76.7%) than women experienced an acute reaction and 35 of them (40.7%) had pathological remote anamnesis positive.

Of the entire study population, 41 patients (47.7%) had cardiac outcomes, 12 patients (13.9%) had gastrointestinal outcomes, 22 (25.7%) had neurological outcomes, 7 patients (8.1%) had renal outcomes, 2 patients (2.3%) had gynaecological outcomes, and 2 patients (2.3%) had dermatological outcomes.

**Figure 3:**
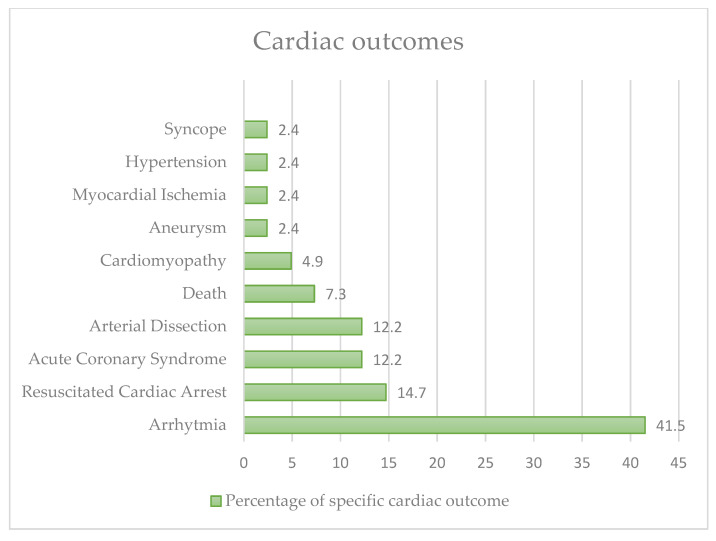
Cardiac outcomes in our review.

Specifically, the cases (*n* = 41) with a cardiac adverse event (Figure 3) were as follows: 17 (41.5%) arrhythmias, 3 (7.3%) deaths, 6 (14.7%) resuscitated cardiac arrests, 1 (2.4%) aneurysm, 5 (12.2%) arterial dissections (aortic or coronary), 2 (4.9%) cardiomyopathies, 5 (12.2%) cases of acute coronary syndrome, 1 (2.4%) case of hypertension, and 1 (2.4%) case of syncope. The median age of patients with cardiological outcomes was 27.7 years. Only in 13 cases (31.7%) was a major pathology found (such as idiopathic QT prolongation, obesity, hypertension, bicuspid aortic valve, dilatation of the ascending aorta, and tetralogy of Fallot). Only nine (21.9%) women had a cardiological outcome.

**Figure 4:**
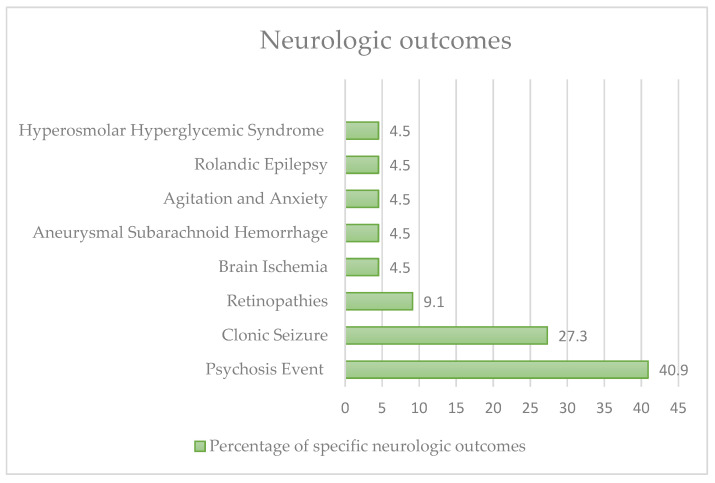
Neurologic outcomes in our review.

A further 22 cases had neurological outcomes (Figure 4): 6 (27.3%) had clonic seizures, 9 (40.9%) experienced a psychotic event, 2 (9.1%) had manifest retinopathies, 1 (4.5%) had cerebral ischaemia, 1 (4.5%) had aneurysmal subarachnoid haemorrhage, 1 (4.5%) had agitation and anxiety, 1 (4.5%) had Rolandic epilepsy, 1 (4.5%) had hyperosmolar hyperglycaemic syndrome with diabetic ketoacidosis. Only 4 cases (18.2%) were women. In 11 cases (50%) there were pre-existing major pathologies (such as schizophrenia, migraine, obesity, hypertension, substance abuse).

**Figure 5:**
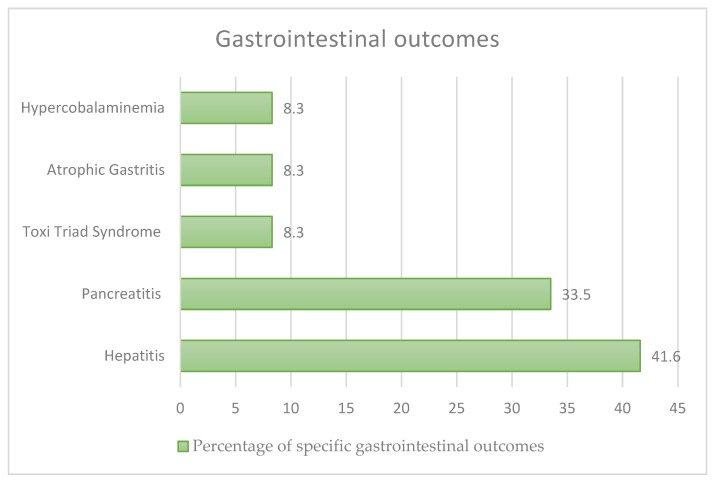
Gastrointestinal outcomes in our review.

There were 12 cases of gastrointestinal problems (Figure 5): 4 (33.5%) had pancreatitis, 5 (41.6%) had hepatitis, 1 (8.3%) had toxic triad syndrome (gastritis, hepatitis and pancreatitis), 1 (8.3%) had hypercobalaminaemia and 1 (8.3%) had atrophic gastritis (AG) and gastrointestinal metaplasia (GIM). Seven cases (58.3%) had major pathologies (such as diabetes mellitus, small-cell left lung carcinoma, serine protease inhibitor Kazal type I (SPINK1) gene mutation, and acute alcoholic pancreatitis). Only 3 (25%) of the 12 cases were female.

**Figure 6:**
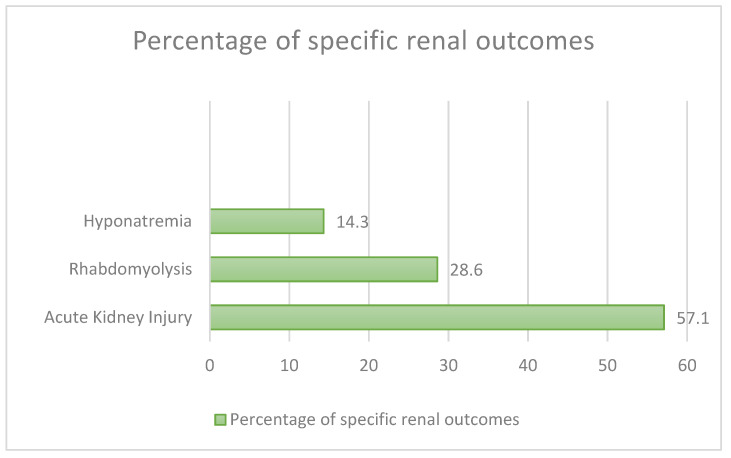
Renal outcomes in our review.

We found seven cases that developed renal disease (Figure 6): four had AKI (57.1%), two (28.6%) had rhabdomyolysis, one (14.3%) had hyponatraemia followed by a coma. Three of these cases (42.8%) had major comorbidities (such as type 2 diabetes mellitus, hypertension, alcohol abuse, PTSD, psychiatric history), and only one of the seven cases (14.3%) was female.

We also had two cases with gynaecological findings; in one case (50%), we found macrosomia and in the other (50%) severe menorrhagia. None of them had major pathologies.

In addition, two cases of erythema have been reported in the scientific literature. One was in a man and the other in a woman. Neither had major pathologies.

There were at least two cases of death. In one case, there was sudden cardiac arrest and in the other case death due to ventricular fibrillation. One was a man and the other was a woman. Only one of them had a major pathology such as mitral valve prolapse.

Nine out of eight-six patients (10%) drank energy drinks with alcohol, one with cannabis, one (1%) with diet pills, one (1%) with another caffeinated drink, and five (6%) took several energy drinks.

**Figure 7:**
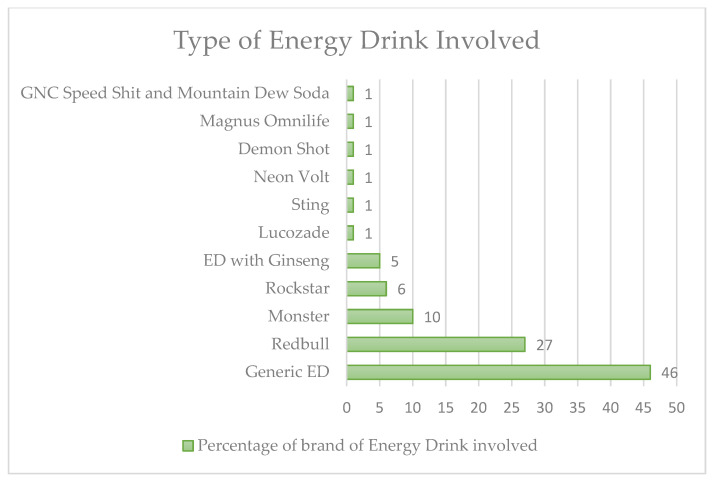
The different types of energy drinks used in our review.

In total, 23 patients (27%) drank Red Bull ED, 9 (10%) drank Monster ED, 4 (5%) drank an energy drink with ginseng, 5 (6%) drank Rockstar ED, 1 (1%) drank Lucozade ED, 1 (1%) drank Sting ED, 1 (1%) drank Neon volt ED, 1 (1%) drank Demon Shot ED, 1 (1%) drank Magnus Omnilife, and 1 (1%) drank GNC Speed Shot and Mountain Dew. Forty patients (46%) drank a generic energy drink (Figure 7).

## 4. Discussion

Consumption of energy drinks has increased in recent years for several reasons [2]. One of the main factors is the aggressive marketing and promotion of energy drinks by beverage companies, primarily targeting young adults and adolescents [101]. This marketing often focuses on the energizing and stimulating effects of energy drinks and their association with extreme sports and other high-energy activities. Another reason for the increasing consumption of energy drinks is the belief that they can improve cognitive and physical performance. Many people consume energy drinks to boost their energy levels, improve focus and concentration, and enhance athletic performance. However, while energy drinks may provide some short-term benefits in these areas, their long-term effects on health and performance remain unclear [102]. Finally, the increasing availability of energy drinks in grocery stores, petrol stations, and other retail outlets has also contributed to their increased consumption. Energy drinks are often promoted as a convenient and portable source of energy and stimulation, making them a popular choice for people who travel a lot or have busy lifestyles. However, the easy availability of energy drinks also means that they are more likely to be consumed in excess, which can increase the risk of negative side effects.

The main psychoactive substance in an energy drink is caffeine. They also contain other ingredients that are thought to increase energy and mental alertness, such as taurine, guarana, ginseng, vitamins, and others [101].

The effects of these drinks on the human body are not fully understood, which is why research into their negative effects has increased.

### 4.1. Effects on the Cardiovascular System

**Figure 8:**
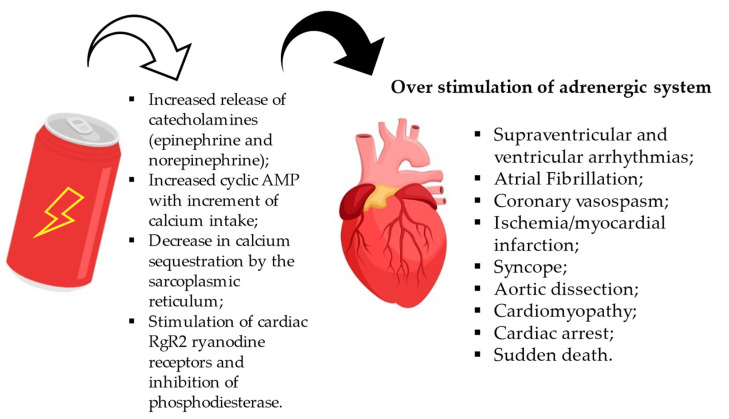
Pathological effects of energy drinks on cardiac tissue.

Effects on the cardiovascular system appear to be the most studied of all the side effects of these substances, due to their potentially fatal properties. The European Cardiac Arrhythmia Society (ECAS) has undertaken a critical review of the reported data on energy drinks, in particular on cardiovascular events and their possible cause–effect relationship, in order to provide recommendations on the safer use of these drinks [103]. High consumption of these energy drinks is associated with an acute haemodynamic and adrenergic state [104], which increases glucose and norepinephrine levels. Supraventricular and ventricular arrhythmias, coronary vasospasm, ischaemia/myocardial infarction, atrial fibrillation, syncope, aortic dissection, cardiomyopathy, cardiac arrest, and sudden cardiac death have been reported in young and otherwise healthy patients [105,106,107,108] (Figure 8). We have found that the risk of cardiovascular outcomes is increased in individuals with pre-existing structural or inherited heart disease. In addition, the consumption of these beverages may lead to the diagnosis of heart disease of which the subjects were previously unaware. Adverse cardiovascular effects have also been found with the use of other substances, such as alcohol. Caffeine [109] has direct chronotropic and positive inotropic effects on the heart. At low concentrations, these effects appear to be due to increased release of catecholamines (epinephrine and norepinephrine) as a result of antagonism of presynaptic receptors for adenosine. At higher concentrations (>10 µM), caffeine can directly increase calcium uptake by increasing cyclic AMP due to inhibition of phosphodiesterase. At very high concentrations (>100 µM) it reduces calcium sequestration by the sarcoplasmic reticulum. At high doses, it induces vascular smooth muscle contraction, except in cerebral vessels. Habitual coffee consumption generally increases peripheral vascular resistance and blood pressure slightly, probably through the release of catecholamines. Systolic and diastolic blood pressure increases by 0.8 mmHg and 0.5 mmHg, respectively, per 100 mg of caffeine. In particularly sensitive individuals, the consumption of a few cups of coffee may cause cardiac arrhythmias, but in most people, parenteral administration of high doses of coffee causes only tachycardia. The stimulation of cardiac RgR2 ryanodine receptors and concomitant inhibition of phosphodiesterase cause a cardio-stimulatory effect, but at high doses, this can cause arrhythmias, tachycardia, and ventricular fibrillation. The ability of caffeine to induce arrhythmias in individuals with atrioventricular conduction disorders or ectopic foci has not been conclusively demonstrated [110]. The inotropic effect of caffeine is enhanced by the positive chronotropic effect of guarana, which contains caffeine, theobromine, and teofiline.

As mentioned above, nine cases of cardiac arrest associated with the consumption of high doses of these stimulants have been reported in literature. The primary triggering mechanism is the occurrence of cardiac arrhythmias such as ventricular fibrillation or the unmasking of previously unrecognised channelopathies. Of the nine cases mentioned above, six required intensive cardiopulmonary resuscitation and no cardiac abnormalities were found in these patients during follow-up visits in the following months, while the other three individuals died (sudden cardiac arrest, STEMI, ventricular fibrillation), but we couldn’t find any available information on their autopsy data in the literature.

### 4.2. Effects on the Neurological System

The consumption of energy drinks containing caffeine and other substances may also have effects on the central nervous system, such as seizures, cerebral vasculopathy and manic psychosis. Studies have shown that these ingredients overstimulate the adrenergic system, leading to hyperglycaemia, hypokalemia, leukocytosis, and metabolic acidosis. The psychostimulant effects of caffeine are evident at low doses.

**Figure 9:**
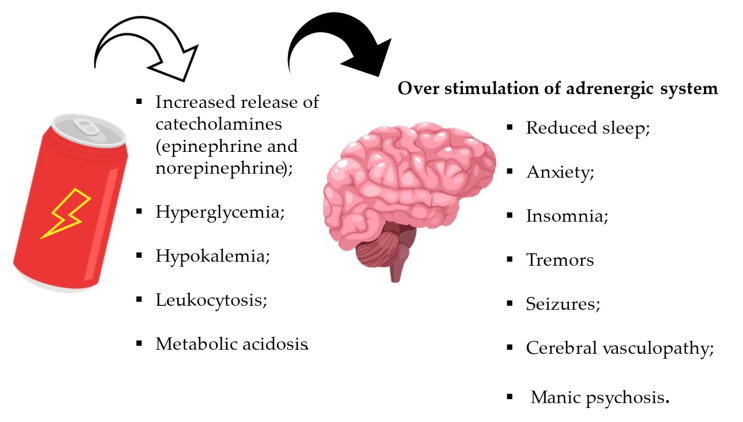
Pathological effects of energy drinks on cerebral tissues.

Caffeine enhances dopamine-related behaviour by inhibiting adenosine A2A receptors and increasing transmission via dopamine D2 receptors. Lorist and Tops [111] used an echoencephalograph (EEG) to highlight the alpha wavelength of the brain (alpha power). They found that caffeine intake increased left frontal activation compared to the right, suggesting that dopamine function may be linked to fatigue, with caffeine reducing fatigue. Doses of less than 500 mg result in increased alertness, increased speed of thoughts and speech, decreased fatigue and reduced sleep. Higher doses may cause restlessness, anxiety, insomnia, tremors, and, in cases of acute toxicity, seizures that do not respond to antiepileptic drugs [112] (Figure 9). The ingestion of caffeine at very high (pharmacological) doses has been associated with the possible occurrence of seizures. In animal models, intraperitoneal administration of caffeine produces convulsions associated with electroencephalography. In humans, seizures have been reported after the overdose or ingestion of drug preparations. The consumption of energy drinks has been associated with the occurrence of seizures, both in patients with known epilepsy and in those without a history of epilepsy [113]. This may be due to the high caffeine content of energy drinks.

At normal average doses of caffeine in humans, caffeine acts as an adenosine receptor antagonist with equal affinity for A1 and A2A receptors. When administered acutely, caffeine acts dominantly on A1 receptors (as ambient adenosine activates them). The chronic use of caffeine leads to the tolerance of A1 receptors. Caffeine then has negligible effects on the A1 receptor and dominant effects on A2A receptors. The endocannabinoids, endogenous ligands of the cannabinoid receptors, are synthesised as needed in response to increased neuronal excitation and activate the presynaptic CB1 receptor, reduce the levels of cyclic AMP (cAMP) released and decrease neurotransmitter release. Caffeine increases neurotransmitter release by removing the inhibitory control of acetylcholine in the hippocampus and prefrontal cortex, regulating the opening of potassium channels mediated by A1 receptors and increasing the firing rate of A2A receptors in the striatum dendritic spines of neurons. This inhibits glutamatergic thalamocortical neurons by inducing cell activation and stimulating the adenylate cyclase pathway. Caffeine blocks A2A receptors and reduces the stimulatory effects of adenosine on cAMP. Caffeine can reduce the inhibition on striatal dopamine transmission by reducing the activity of striatal neurons and causing the disinhibition of thalamo-cortical projection neurons. The activation of A2A receptors leads to cAMP production, and the activation of D2 receptors reduces cAMP production and causes an inverse regulation of the activity of cAMP-dependent protein kinase (PKA) [114]. As caffeine mimics the effect of dopamine on striatopallidal neurons, it causes a progressive sensitisation of cannabinoid CB1 receptors, which control GABAergic inhibitory postsynaptic currents (IPSCs) [115]. The caffeine blockade of A2A receptors reduces the activation of cAMP-PKA pathways, resulting in increased glutamate release, the activation of mGlu5 metabotropic receptors, and endocannabinoid release. The blockade of adenosine A2A receptors in the striatum has been linked to the psychoactive properties of caffeine. There is also evidence that a specific genetic polymorphism of the adenosine A2A receptor influences habitual caffeine consumption in humans [116].

Richard and Smith [117] recently reviewed the literature on the chronic mental health effects of energy drinks. They concluded that while the acute effects of energy drinks on mood appear to be positive, chronic consumption is associated with stress, anxiety, and depression. Taurine is a molecule that crosses the blood–brain barrier and binds to GABA receptors. It can mimic the effects of GABA and glycine, resulting in an anticonvulsant effect that has a stabilising effect on membranes inside and outside the cell.

### 4.3. Effects on the Gastrointestinal and Renal System

**Figure 10:**
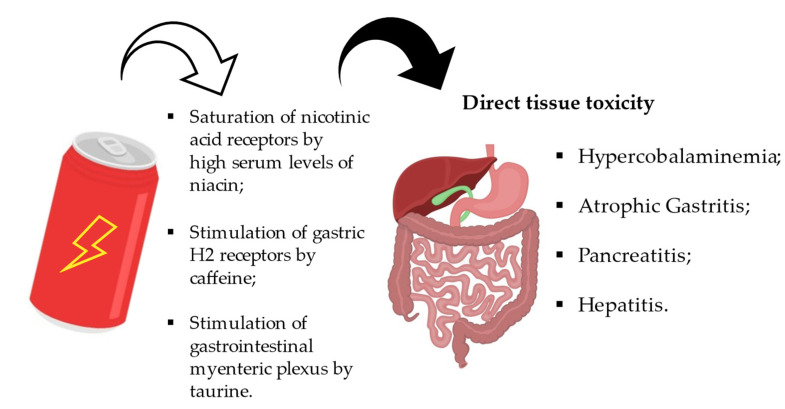
Pathological effects of Energy Drinks on gastrointestinal tissues.

These drinks can also lead to the development of gastrointestinal and renal disorders. Some authors describe cases of acute hepatitis, acute pancreatitis, and renal failure with acute kidney injury (AKI). As mentioned above, all energy drinks contain high doses of caffeine, taurine, sugar, and vitamins. A megadose of vitamin B3 (niacin) is associated with hepatotoxicity. Niacin hepatotoxicity is thought to be a dose-dependent, directly toxic response. Vitamin B3 is associated with cellular metabolism and flushing and hepatotoxicity at pharmacological doses. Hepatotoxicity manifests as a mild elevation of liver enzymes (ALT/AST), hepatic steatosis, hepatic necrosis, and, in rare cases, liver failure. The lowest dose of vitamin B3 known to cause hepatotoxicity, as reported in the literature, is 1 g/day [31] (Figure 10). However, the main cause of AKI was most likely taurine, which is used as a dietary supplement by athletes to enhance performance.

**Figure 11:**
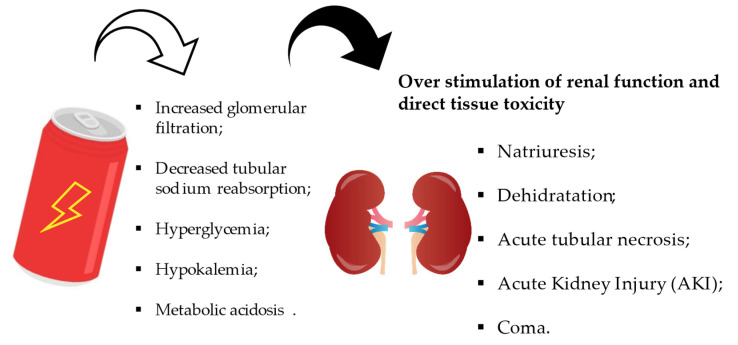
Pathological effects of energy drinks on renal tissue.

Caffeine promotes digestion by stimulating salivation and gastric juice production due to the presence of synergistic substances acting on H2 receptors. Caffeine is also known to relax the gastroesophageal sphincter, which prevents the stomach contents from rising into the oesophagus. In addition to its renal effects, caffeine is a weak diuretic. This effect may be associated with an increase in glomerular filtration and a decrease in tubular sodium reabsorption (Figure 11). There are also GABA receptors in the gastrointestinal tract. These are located in the peripheral autonomic nervous system and are involved in acid secretion and the protection of the gastric mucosa from injury and motility. In the stomach, taurine accumulates in the parietal cells of the gastric glands. Taurine-containing cells are found in the myenteric plexus and submucosal plexus of the enteric nervous system. The taurinergic neurons in the muscle layer of the gastrointestinal tract and the gastrointestinal tract may be involved in gastrointestinal motility and endocrine cell functions [118].

### 4.4. Other Effects

Finally, there are rare cases of obstetric, dermatological, and autoimmune complications that are difficult to explain or whose association with energy drinks does not seem to be reliable and well explained. Regarding autoimmune and skin complications, some authors are convinced that there are underlying mechanisms of hypersensitivity to synthetic taurine, which may be slightly different from natural taurine [119]. Although the authors have not been able to elucidate the mechanism of anaphylaxis, they suggest that the additives used to stabilise the amino acids, such as sulphites, butylated hydroxyanisole, butylated hydroxytoluene, and olysorbate emulsifier, may be the cause of the symptoms [120]. There is also no clear association with energy drinks in obstetric complications. We reported two cases of such complications. In the first case, we had neonatal hyperinsulinism due to isolated high maternal sugar intake, an event that has never been reported in the literature. In the second case, we had menorrhagia due to secondary VKD (acquired vitamin K deficiency) after consumption of high-energy drinks. There is no evidence in the literature of ingredients in energy drinks that might correlate with the development of VKD.

### 4.5. Experimental Studies on Animal Models

The adverse health effects have also been studied by various research groups using animals as an experimental model. The summarised results of these studies, outlined below, have shown effects similar to those observed in humans, with alterations affecting various organs or systems.

Salih et al. [121] used rabbits as an animal model to observe the histopathological effects of energy drinks (EDs) on various organs, including the brain, liver, kidneys, and heart. Their findings suggest a direct correlation between tissue damage and the dose administered. At higher doses, they observed renal vascular congestion, the bleeding of interstitial tissue, focal atrophy, and the degeneration of the lining epithelium of the proximal and distal convoluted tubules. Nieradko Iwanicka and colleagues [122] studied the effects of the ad libitum consumption of energy drinks in mice on memory, body weight and laboratory parameters. After 30 days of the experiment, the researchers observed weight gain in male mice, an increase in serum transaminases and cholesterol concentration, but no memory-related changes. Similar results were found by Sadowska [123], who studied the effects of energy drink consumption in 30 mice and highlighted three main consequences: reduced body weight gain despite increased energy expenditure, suggesting an increased catabolic rate in the animals studied; reduced peri-intestinal fat deposition and increased accumulation of peri-cardiac adipose tissue, which may act as a source of chemokines and cytokines with pro-inflammatory properties. Finally, energy drink consumption led to an increase in blood glucose concentration, most likely due to metabolic changes leading to increased lipolysis and the development of insulin resistance.

Rasheed and colleagues [124] investigated the effects of energy drinks on renal tubules using albino rats as an experimental animal model. Their research showed histopathological changes in renal tubular cells, such as increased tubular vacuolisation, in rats exposed to energy drinks. According to the researchers, this adverse effect is due to inhibition of the A2A adenosine receptor, resulting in increased oxidative stress and production of inflammatory stimuli.

Abonar et al. [125], Rehman et al. [126], and Haroun et al. [127] investigated the effect of energy drinks on the pancreas of adult male albino rats. They performed histological, immunohistochemical and biochemical studies that revealed alterations in pancreatic cytoarchitecture. Specifically, damage to the pancreatic acini and islets of Langerhans was observed, accompanied by an increase in collagen deposition in the pancreatic parenchyma, a decrease in serum insulin levels, and an increase in blood glucose levels. There was also an increase in TNF-a, NO, and malondialdehyde levels, indicating a global negative effect on both exocrine and endocrine functions of the gland. Kassab et al. [128] also studied the effects of energy drinks on the salivary glands of thirty adult albino rats and observed parenchymal changes, including cytoplasmic vacuolisation, pyknotic nuclei and abundant collagen fibre deposition, resulting in the displacement of striated muscle fibres. However, these pathological changes in the glands were found to be transient upon cessation of the substance.

Possible adverse effects of caffeine and taurine on cardiac electrophysiology were investigated using twenty-five rabbits as an animal model [129]. The hearts of animals perfused with caffeine and taurine showed shortened repolarisation times and refractory periods on the ECG trace, followed by ventricular arrhythmias, confirming the potential arrhythmogenic effect of these substances. In another study, Demirel et al. [130] investigated the effects of the combined consumption of energy drinks and alcohol on the myocardium and skeletal muscle system. In particular, the study highlighted damage to the cardiac and endothelial cytoarchitecture, as well as an increased tendency towards anaerobic cellular respiration in skeletal muscle tissue, resulting in increased lactate formation. Diaz et al. [131] also investigated the effects of these two substances in combination. In their experimental study of rats exposed to the substances for 90 days, changes in the temporal cortex and hippocampus were analysed. The results show an inflammatory response associated with oxidative stress, local gliosis, and increased levels of IL-1, TNF-1, iNOS, reactive oxygen species, lipid peroxidation, and nitric oxide. In addition, at the neurological level, Ulenius [132] and colleagues demonstrated that the combination of caffeine and taurine enhances the stimulant properties of ethanol on the locomotor system, a phenomenon previously associated with substance dependence and associated with increased dopamine levels and reward circuits. Ugwuja [133] conducted experiments to assess the biochemical effects of energy drinks alone or in combination with alcohol on albino rats. The study showed changes in total white blood cell count, plasma potassium, calcium, renal function, liver enzymes, and plasma triglycerides. Krahe et al. [134] also analysed the effects of combined energy drink and alcohol consumption. Overall, animals treated with alcohol and energy drinks showed increased locomotor activity and increased anxiety levels in the open field test. They also showed an early loss of the righting reflex and poorer motor coordination in the rotarod test. These effects on righting reflex and motor coordination were associated with the over-activation of cerebellar GABAA receptors. The data also show that exposure to alcohol in combination with energy drinks prolongs the duration of motor impairment and ataxia in adolescent mice. This ability to prolong the effects of alcohol may explain why this group performed worse in the righting reflex loss test after cumulative administration of alcohol and energy drinks compared to animals receiving alcohol alone. Reis et al. [135] investigated the effects of 14 days of energy drink consumption alone or in combination with ethanol on oxidative stress parameters, including superoxide dismutase (SOD), catalase (CAT), glutathione peroxidase (GSH-Px), and the lipid peroxidation marker malondialdehyde (MDA) in 40-day “adolescent” mice. The ethanol-treated group showed a significant increase in SOD and GSH-Px activity in brain tissue compared with the control group. The elevated MDA levels observed in rats co-exposed to energy drinks and ethanol, as well as those exposed to energy drinks alone, may be a consequence of increased free radical formation and altered cellular antioxidant defence status. Liver histopathology results show that energy drinks may induce liver damage, and the combined effect of ethanol and energy drinks may cause more significant damage than either substance alone, as indicated by increased MDA levels. A histopathological examination of brain tissue did not show any treatment-related abnormalities, possibly due to the short duration of the experiment.

Nasi and colleagues [136] investigated the possible negative effect of energy drinks on the gastrointestinal tract by administering different substances to rats for five consecutive days. They did not observe any acute lesions in the gastrointestinal tract, but they did observe an eosinophilic infiltration in the intestinal mucosa. This histopathological change was also observed in rats treated with caffeine alone, suggesting that this inflammatory effect is a direct consequence of this substance, which is also present in energy drinks.

Elçi et al. [137] investigated the effects of eight weeks of energy drink consumption on the female reproductive system, specifically analysing follicular ovarian reserve and anti-Müllerian hormone levels in the blood. They found a significant decrease in both parameters. Instead, Oyelowo et al. [138] focused on the biochemical effects of both natural and artificial energy drinks on testicular tissue after 28 consecutive days of consumption in pubertal male rats. Their results showed negative effects of energy drink consumption, whether natural or artificial, on male reproductive functions, including decreased testosterone steroidogenesis in Leydig cells, changes in gonadotropin synthesis, and disruption of sperm homeostasis.

Al-Basher et al. [139] conducted a study on the effects of perinatal exposure to caffeine-based energy drinks on the liver, kidneys, brain, locomotor activity, and anxiety in newborn mice. Pregnant mice received 2.5 or 5 mL of energy drinks from the first day of pregnancy until 15 days after birth. Perinatal exposure to energy drinks resulted in a significant increase in lipid peroxidation (MDA) and a decrease in antioxidant defences in the liver, kidneys, brain, cerebellum, and medulla oblongata of newborn mice on days 21 and 35 after birth. Energy drinks also induced various histological alterations, including vacuolation and lipid infiltration of hepatocytes, developing degenerated glomeruli and dilated interstitial spaces in the renal cortex, pyknosis and chromatolysis of cerebral and medullary neurons, and degenerated and abnormal Purkinje cells in the cerebellum. In addition, energy drinks increased locomotor activity and induced anxiety-like behaviour in newborn mice.

Posokhov et al. [140] investigated the effects of two months of energy drink consumption on red blood cell membranes. They used the fluorescent probe O1O (2-(2′-OH-phenyl)-5-phenyl-1,3-oxazole), which localises to the area of glycerol backbones, carbonyl groups of phospholipids, and hydrocarbon chains of phospholipids (near carbonyl groups) in the bilayer. The consumption of energy drinks was associated with increased fluorescence intensity in erythrocyte suspensions compared to control animals. The observed change in probe fluorescence is attributed to an increase in the viscosity of the probe environment within the membrane. Using the fluorescent probe O1O, it was shown that the long-term oral administration of caffeine-based energy drinks to rats caused an increase in membrane viscosity (resulting in reduced fluidity) in red blood cells.

## 5. Conclusions

This extensive literature review includes a large number of research studies on the potentially fatal health effects of both acute and chronic abuse of these substances. These consequences include cardiac arrhythmias, neurological and behavioural changes, acute organ inflammation (including the liver, stomach, pancreas, and kidneys) and even cases of rare dermatitis or autoimmune disorders. Furthermore, although based on a limited case pool, it is noteworthy that there is a marked disparity in the literature between cases of cardiac arrest requiring intensive cardiopulmonary resuscitation (nine cases) and documented deaths (three cases) resulting from energy drink abuse. These statistics suggest a plausible under-reporting of deaths associated with these substances, particularly among frequent users such as adolescents and athletes. Consequently, in the investigation of sudden cardiac death in young people, the role of the pathologist in meticulously collecting anamnestic and circumstantial data from the deceased, recognising the potential involvement of non-illicit substances such as energy drinks, becomes crucial [141].

The results of experimental studies in animal models echo the findings of the review, demonstrating acute and chronic effects consistent with observations in humans.

Although individual components have been shown to be safe [142], excessive consumption, especially among adolescents, often leads to potential adverse effects on human health. As shown in this review, these effects can vary, particularly regarding the cardiovascular and cerebral systems. It would therefore be important to consider the introduction of precise limits on the consumption of these drinks. As caffeine is the most representative ingredient in terms of composition, it is first necessary to consider the upper limits of safe caffeine intake. Most cans of energy drinks (250 mL) contain 50 to 150 mg of caffeine, while the EFSA upper safe intake limit for adults is up to 400 mg per day (about 5.7 mg/kg bw per day for a 70 kg adult), with a single dose not exceeding 200 mg [143]. In fact, no health concerns regarding acute toxicity, bone status, cardiovascular health, cancer risk, or male fertility have been raised by other agencies in previous assessments at this level of habitual caffeine consumption. The FDA (Food and Drug Administration) estimates that toxic effects, such as seizures, may be observed following the rapid consumption of about 1200 mg of caffeine, or about 0.15 tablespoons of pure caffeine [144]. For pregnant or breastfeeding women, the safe daily intake of caffeine is halved from 400 mg to 200 mg, or about half a can. Finally, for children and adolescents, it is important to accentuate that neither EFSA nor the FDA have indicated a safe limit, suggesting that these substances should be avoided altogether, as further emphasised by the American Academy of Pediatrics due to possible long-term negative effects on behavioural disorders.

Therefore, based on our observations and those found in the literature, we suggest that the daily intake of energy drinks should not only not exceed the safety limits for caffeine established by European and American regulatory authorities, but should be even lower. Indeed, these drinks also contain other neurostimulants, the effects of which are not fully understood. Furthermore, as this review points out, there are cases in the literature of people with no known medical conditions who have suffered acute cardiac events after consuming just a few 250 mL cans of these drinks. Given that the concentration of caffeine in these drinks is between 50 and 150 mg per can (250 mL), we recommend no more than one can at a time and two cans per day to remain within an acceptable safety limit. We also believe that it is necessary to clearly state the daily intake limit for products containing high levels of caffeine (such as ‘Demon Energy Shot’, which contains 200 mg of caffeine in 60 mL of product), given the potential risk of acute caffeine intoxication [145].

In addition, the sale and consumption of these drinks in minors should be regulated as, although they are legal substances, their long-term effects are not yet known and may lead to psychiatric pathologies or the aggravation of cardiac conduction disorders. Increased public education on the potential risks associated with the misuse of energy drinks is warranted to enable individuals to make informed decisions regarding consumption.

Furthermore, extensive research is needed to elucidate the long-term effects of energy drink consumption on human health.

## Figures and Tables

**Table 2:** The results of our review on cardiac side effects.

References	N° Cases	Age/Sex	Energy Drink (If Applicable) or Substances	Major Pathology	Onset	Adverse Event
Argano et al., 2011 [27]	1	38/M (Male)	Red Bull	N/A [not available]	Consumption of 5 cans of Red Bull per day 4 days before the onset of symptoms	Haemorrhagic aneurysm of the anterior communicating artery
Avci et al., 2013 [28]	1	28/M	ED [generic energy drink]	N/A	Consumption of 3 cans of 250 mL energy drink 5 h before a basketball game. Taking one ED a day for 7 months	Death from sudden cardiac arrest.
Berger et Alford, 2009 [29]	1	28/M	ED	N/A	Ingestion of 7–8 cans of a caffeinated “energy drink” between 8 am and his collapse 7 h later	Resuscitated cardiac arrest with anteroseptal ST Elevation Myocardial Infarction (STEMI).
Benjo et al., 2012 [30]	1	24/M	ED + vodka	N/A	Symptoms started 1 or 2 h after drinking vodka mixed with an energy drink.	Left main coronary artery acute thrombosis.
Cannon et al., 2001 [31]	1	25/F (Female)	Race 2005 Energy Blast with Guarana and Ginseng	Mitral valve prolapses	Consumption of a 55 mL shot of “Race 2005 Energy Blast with Guarana and Ginseng” before onset.	Death after ventricular fibrillation.
Choudhury et al., 2017 [32]	1	53/M	Red Bull	N/A	Ingestion of 2 cans of Red Bull shortly before the test, thinking it would improve his performance on the treadmill.	Partial right bundle branch block, hypertension.
Di Rocco et al., 2016 [33]	2	14/M	Red Bull + caffeinated drink	N/A	Intake of an unknown amount of a highly caffeinated drink the day before a race. He also reported drinking a Red Bull™ energy drink five days prior to enrolment.	Arrhythmia
		16/M	Red Bull + vodka	Attention deficit hyperactivity disorder, asthma, and allergies	Ingestion of an unknown quantity of Red Bull™ mixed with vodka at a party.	Atrial tachycardia/atrial Fibrillation (AF)
Dufendach et al., 2012 [34]	1	13/F	ED	Idiopathic QT prolongation	Consumption of at least one 16 oz (476 mL) can of an energy drink (160 mg caffeine) before onset.	Palpitations and chest pain, associated with a QTc
Enriquez et Frankel, 2017 [35]	2	19/F	Monster ED	N/A	Consumption of three 8 fl oz (263 mL) cans of Monster ED within 2 h of onset.	Resuscitated cardiac arrest.
		23/F	Red Bull	Peripartum cardiomyopathy, subcutaneous ICD (Implantable Cardioverter Defibrillator), left ventricular ejection (20%)	Ingestion of a can of Red Bull for the first time in her life	Syncope
Gharacholou et al., 2017 [36]	1	27/M	Rockstar energy	Family history of coronary artery disease.	Ingestion of 4–5 beverages in 12-h.	Acute anterior ST-segment elevation myocardial infarction (STEMI)
Hanif et al., 2020 [37]	1	22/M	ED	N/A	Consumption of two energy drinks (111 mg of caffeine and taurine) before onset	Atrial fibrillation
Israelit et al., 2012 [38]	1	24/M	ED	Obesity, hypertension.	Ingestion about 20 cans of energy drink (XL) over the previous night	Death after ST elevation myocardial infarction (STEMI)
Jonjev et Bala, 2013 [39]	3	54/M	Red Bull	Obesity, hypertension	Consumption of 4–5 high-energy drinks (mostly Red Bull) per night.	Subacute aortic dissection (De Bakey type I)
		26/M	ED	Bicuspid aortic valve, dilation of the ascending aorta	Consumption of 5–6 high-energy drinks per night just after a party	Acute aortic dissection (De Bakey type II)
		48/M	ED	Hypertension, myocardial infarction.	Consumption of high energy drinks before onset.	Acute aortic dissection (De Bakey type I)
Kaoukis et al., 2012 [40]	1	24/M	ED	N/A	Ingestion of a small amount of an energy drink in little cups one after another.	Reverse Takotsubo Cardiomyopathy
Khan et al., 2015 [41]	1	27/M	Red Bull	PVCs (Premature ventricular contractions)	Ingestion of 6 red bull energy drinks per day for over 6–8 months.	Resuscitated cardiac arrest with ventricular fibrillation
Mattioli et al., 2018 [42]	3	22/M	ED	N/A	Consumption of 750 mL of ED	Atrial fibrillation (AF)
		23/M	ED	N/A	Consumption of 600 mL of ED	Atrial fibrillation (AF)
		26/M	ED + alcohol	N/A	Consumption of an alcoholic beverage, corresponding to 30 g of alcohol, associated with 600 mL of EDs	Atrial fibrillation (AF)
Nagajothi et al., 2008 [43]	1	23/F	GNC Speed Shot and Mountain Dew soda drink	N/A	Ingestion of one can of GNC and one of Mountain Dew (containing caffeine) before onset	Supraventricular tachycardia
Osman et al., 2019 [44]	1	32/M	ED	N/A	Consumption of 48 cans of 250 mL ED (XXL, containing a mixture of caffeine, vodka, and taurine) over the past three days.	Ventricular fibrillation
Peake et al., 2007 [45]	1	58/m	ED	N/A	Ingestion of one bottle (1000 mL) per week of a highly caffeinated (caffeine content 4.04 mg/mL) commercially available energy drink for 6 months	Atrial fibrillation-related cardiomyopathy
Polat et al., 2013 [46]	1	13/M	ED	N/A	Consumption of an energy drink for the first time the night before. About 8 h after consuming the energy drink, the patient’s chest pain started.	Spontaneous coronary artery dissection (SCAD)
Roettlaender et al., 2012 [47]	1	22/F	ED	N/A	Consumption of six cans of a caffeinated energy drink within 4 h	Resuscitated cardiac arrest due to long QT syndrome.
Rutledge et al., 2012 [48]	1	24/M	Red Bull + vodka	Unknown Brugada Syndrome	Consumption of a Red Bull energy drink (80 mg caffeine and 1000 mg taurine) combined with vodka.	Resuscitated cardiac arrest.
Sattari et al., 2016 [49]	1	28/M	Monster ED	N/A	Ingestion of two Monster ED, two to three beers, and chewing tobacco for several months.	AF
Scott et al., 2011 [50]	1	19/M	Red Bull	Gastro-oesophageal reflux disease	Ingestion of around two to three cans of ‘Red Bull’ daily	STEMI
Solomin et al., 2015 [51]	1	26/M	Monster ED + Rockstar energy	N/A	Consumption of any kind of energy drink: eight to ten 473 mL drinks per day	STEMI
Ten Bos et al., 2016 [52]	1	45/F	Red Bull	N/A	High consumption of Red Bull energy drinks.	Myocardial ischaemia
Terlizzi et al., 2008 [53]	1	16/F	Red Bull	N/A	Consumption of four to five cans before onset.	Reversible postural tachycardia syndrome
Torbey et al., 2011 [54]	1	43/F	Panax ginseng + ED	N/A	Consumption of at least 70 cc of caffeine and 4 L of Korean Panax ginseng daily for six months.	Long QT syndrome.
Ullah et al., 2018 [55]	1	25/M	ED	N/A	Drinking large amounts of caffeinated ED every day for the past week.	Acute coronary syndrome
Unal et al., 2014 [56]	1	32/M	ED	N/A	Consumption of 5 bottles of energy drink before onset.	Left main coronary artery thrombosis and acute anterior myocardial infarction
Usman et Jawaid, 2012 [57]	1	16/M	“Sting” energy drink	N/A	Consumption of about 80–100 cans in the past 2 weeks, an average of 3 cans per day.	Hypertension.
Uyanik et al., 2021 [58]	1	24/M	ED	N/A	Ingestion of 8 to 10 cans of energy drinks per day (3.5–4 L/day) in the past 2 weeks	Cardiomyopathy
Ward et al., 2014 [59]	1	45/M	Red Bull	Tetralogy of Fallot	Consumption of 3 Red Bull energy drinks before onset.	Resuscitated cardiac arrest with ventricular tachycardia.
Wilson et al., 2012 [60]	1	17/M	Red Bull + Monster	Myopericarditis	Ingestion of caffeinated energy drinks (3–4 Red Bull 80 mg of caffeine/can and 2–3 Monster 160 mg of caffeine/can) before onset.	Coronary artery vasospasm
Zacher et al., 2018 [61]	1	25/M	ED + alcohol	N/A	Ingestion of 8 cans, mixed with strong liquor, before onset.	Spontaneous coronary artery dissection

**Table 3:** The results of our review on gastrointestinal side effects.

References	N° Cases	Age/Sex	Energy Drink (If Applicable) or Substances	Major Pathologies	Onset	Adverse Event
Abdisamad et al., 2019 [62]	1	46/M	ED	Diabetes mellitus	Daily consumption of 3–4 16 oz cans for the 2 weeks before onset.	Acute pancreatitis, hepatomegaly, hypertriglyceridemia.
Al Yacoub et al., 2020 [63]	1	62/F	Sugar-free ED	Small-cell left lung carcinoma.	Ingestion of five to six cans of a 16 fluid oz sugar-free ED daily.	Acute hepatitis.
Al-Tamini et al., 2018 [64]	1	19/M	Full Throttle + Red Bull	Serine Protease Inhibitor Kazal-type I (SPINK1) gene mutation.	Regular consumption of Full Throttle and Red Bull.	Acute pancreatitis
Apestegui et al., 2011 [65]	1	16/M	Red Bull	Biliary tumour; retransplantation	Consumption of 3 cans of Red Bull in 4 h to stay fit for his examinations. The next day, he separately took 2 tablets (800 mg) of the non-steroidal anti-inflammatory drug ibuprofen for a headache.	Acute hepatitis
Garg et al., 2020 [66]	1	34/F	Red Bull and Monster Energy	N/A	Regular use of 1–2 ED/day for the past 15 years.	Atrophic gastritis (AG) and Gastric intestinal metaplasia (GIM).
Harb et al., 2016 [67]	1	50/M	ED	N/A	Ingestion of 4–5 ED before onset.	Acute hepatitis.
Huang et al., 2014 [68]	1	36/M	Rockstar energy	N/A	Consumption of 3 Rockstar energy drinks per day for the past year.	Acute hepatitis.
Randhawa et al., 2022 [69]	1	29/M	ED	N/A	Consumed 5 to 6 energy drinks a day. Consumed 7 16-ounce energy drinks the day before hospital admission.	Acute pancreatitis.
Shmelev et al., 2015 [70]	1	40/M	Rockstar energy	Acute alcoholic pancreatitis with pseudocyst formation.	Consumption of Rockstar™ followed by unexplained episodes of pancreatitis	Acute pancreatitis.
Takahashi et al., 2013 [71]	1	76/M	ED	Hypertension; benign prostatic hypertrophy; total gastrectomy	Drinking half a bottle of energy drink as a dietary supplement after total gastrectomy.	Hypercobalaminemia.
Uwaifo, 2019 [72]	1	46/M	Monster energy	Type 2 diabetes, nephrectomy, hyperuricemia.	Daily consumption of 2–3 16 oz cans for 4 months	Toxic triad syndrome of gastritis, hepatitis and pancreatitis.
Vivekanandarajah et al., 2011 [73]	1	22/F	ED	N/A	Daily ingestion of 10 cans of an energy drink.	Acute hepatitis.

**Table 4:** The results of our review on neurological side effects.

References	N° Cases	Age/Sex	Energy Drink (If Applicable) or Substances	Major Pathologies	Onset	Adverse Event
Casas-Gomez et al., 2018 [74]	1	22/M	ED	N/A	Ingestion of 20 cans of 250 cc of an ED in 24 h because he was worried about flying.	Maniac episode.
Calabrò et al., 2012 [75]	1	20/M	Red Bull	N/A	Consumed 4 to 6 cans of Red Bull a day for about 5 months before onset of seizures.	Tonic–clonic seizure.
Cerimele et al., 2010 [76]	1	43/M	ED	Schizophrenia, paranoid type, and alcohol dependence in remission	Consumption of energy drinks 8 weeks prior to presentation. After drinking his first can of the drink, he decided to increase his daily consumption to 8 to 10 cans (16 oz per can) a day.	Paranoia, internal preoccupations, constricted affect, and delusional religious beliefs.
Cruzado et al., 2014 [77]	1	31/F	Energy drink Magnus Omnilife products (each ration contains 81 mg of caffeine, 202 mg of taurine and 151 mg of glycine).	N/A	Increase consumption of filtered coffee from 2 to 5 cups a day and drink 3 to 4 rations of the Magnus Omnilife Products energy drink a day. After a few days, up to 10 daily portions of the energy drink (in addition to the five cups of filtered coffee per day).	Delusional ideas of grandeur, auditory hallucinations, and lacked awareness of disease manic syndrome.
Gorgulu et al., 2014 [78]	1	21/M	ED + vodka	N/A	Regularly consumed one or two energy drinks a day in order to be more energetic during his training, sometimes drank energy drink in combination with a small amount of vodka.	Hallucinations, disorganized behavior, excitation, persecution, mystic and grandious delusions.
Grant et al., 2016 [79]	1	44/F	Monster Energy drink	N/A	Consumption of five oversized cans (16 oz/can) of Monster Energy drink (totalling 800 mg of caffeine)	Aneurysmal subarachnoid haemorrhage complicated by severe catheter-induced vasospasm and symptomatic thromboembolism.
Gupta et al., 2019 [80]	1	34/F	Multiple ED	N/A	Consumption multiple energy drinks before onset.	Macular neuroretinopathy (AMN)
Hernandez-Huerta et al., 2017 [81]	1	18/M	ED + cannabis	Daily use of tobacco, daily use of cannabis. A paternal uncle had an unspecified chronic mental illness.	Consumption of 6 ED cans (80 mg caffeine per can) per day during in the last seven days before onset.	Psychotic disorder
Iyadurai et Chung 2007 [82]	4	25/M	Rockstar energy	N/A	Drink two 24 oz bottles of Rockstar energy drink on an empty stomach about 30 to 60 min before the seizure.	Tonic–clonic seizure
		19/M	ED	Complex migraine	Ingestion of energy drinks on an empty stomach approximately 2 h prior to each ‘‘seizure’’ episode.	Tonic–clonic seizure
		28/F	Monster energy + diet pills	Migraine	Regular consumption of an energy drink (Monster).	Tonic–clonic seizure
		26/M	Monster energy	N/A	Ingestion of more than 2 24 oz cans before onset.	Tonic–clonic seizure
Kelsey et al., 2019 [83]	1	30/M	Red Bull + alcohol	One episode of low mood 12 years prior, and one deliberate over-dose a year later.	Consumed up to twelve 250 mL cans of Red Bull per day. Consumed 7 units of alcohol on the day of arrest.	Agitation and anxiety
Laiseca et al., 2018 [84]	1	8/M	Monster energy	N/A	Ingestion of energy drinks in the previous week (500-mL cans of Monster Energy).	Rolandic epilepsy
Menkes, 2011 [85]	1	27/M	Demon Shot energy drink	Obesity (125 kg), schizophrenia	Consumed a 60 mL Demon Shot energy drink and enjoyed an hour-long “buzz”. Repeating the dose did not produce the desired effect. One week later, three shots over 15 min.	Transient psychotic
Norelli et Xu, 2014 [86]	2	23/M	Red Ginseng drink	N/A	Consumption of seven times the recommended dose of ginseng every day for months.	Manic psychosis
		79/M	Red Ginseng drink	Substance-induced hypomanic episode	Ingestion of Korean red ginseng 3–4 times per day, everyday, during the two months prior the episode	Hypomanic psychosis
Pagano et al., 2017 [87]	1	48/M	ED	Hypertension	Consumption of three large cans (473 mL each) of an energy drink over 30–40 min before onset.	Deep intraretinal haemorrhages
Sharma, 2010 [88]	1	32/M	Red Bull	Occasional ‘mood swings’, alcohol abuse. Family history: post-partum depression and a case of suicide.	Consumption of 6–8 large cans (550 mL per can) a day during the week preceding his hospitalisation.	Substance-induced mood disorder with maniac features.
Staikoglou et al., 2022 [89]	1	14/M	ED	N/A	Consumption of 2 L of energy drink during the previous 10 h before onset.	Brain ischemia secondary to dissection of the posterior cerebral artery (PCA)
Trabulo et al., 2011 [90]	1	28/M	Red Bull	Heroin and cocaine abuse, chronic hepatitis C, severe mitral insufficiency and post-infectious endocarditis.	Ingestion of several cans (about 6) of Red Bull together with coffee in the 4 h before onset.	Clonic seizures + tonic clonic seizures
Yartsev et Peisah, 2021 [91]	1	35/M	Red Bull	Chronic schizophrenia, obesity (142 kg), childhood asthma, gastro-oesophageal reflux disease, hypercholesterolaemia and impaired glucose tolerance	Consumption of energy drinks for weight loss, with daily caffeine consumption varying between 150 and 450 mg/day (between two and six 250 mL cans of Red Bull per day, with a caffeine concentration of 30 mg/100 mL).	Hyperosmolar hyperglycaemic syndrome with diabetic ketoacidosis.

**Table 5:** The results of our review on renal side effects.

References	N° Cases	Age/Sex	Energy Drink (If Applicable) or Substances	Major Pathologies	Onset	Adverse Event
Al Yacoub et al., 2020 [63]	1	62/F	Sugar-free ED	Small-cell left lung carcinoma.	Consumption of five to six cans of a 16 fluid oz sugar-free ED daily.	Acute kidney injury (AKI)
Greene et al., 2014 [92]	1	40/M	Red Bull	Diabetes mellitus type 2, hypertension, anxiety, depression, alcohol abuse, posttraumatic stress disorder, chronic obstructive pulmonary disease, obstructive sleep apnoea, gout, and hypertriglyceridemia-induced pancreatitis.	Ingestion of at least 5 to 6 20 oz (590 mL) Red Bull energy drinks daily for several weeks.	AKI
Icin et al., 2017 [93]	1	27/M	ED	N/A	Consumption of 5000 mL of beer, 400–500 mL of liquor, and 2000 mL of energy drink (containing 25 mL/100 mL of caffeine and taurine, vitamins, sugar, citric acid, and caramel) during a 6-h period before the onset of symptoms.	Hyponatremia followed by coma
Iyer et al., 2016 [94]	1	35/M	“Neon Volt”	N/A	Consumed 2 bottles of Neon Volt and then started training	Rhabdomyolysis
Kelsey et al., 2019 [83]	1	30/M	Red Bull + alcohol	The psychiatric history consisted of a depressive episode 12 years earlier and a deliberate overdose one year later. On both occasions, no referral to psychiatric services was made and no treatment was initiated.	Consumption of up to twelve 250 mL cans of Red Bull per day, and he also had consumed 7 units of alcohol on the day of his arrest.	AKI
Schoffl et al., 2011 [95]	1	17/M	ED + vodka	N/A	Ingestion of 3 L of ED with 1 L of vodka before onset	AKI

**Table 6:** The results of our review on gynaecological side effects.

References	N° Cases	Age/Sex	Energy Drink (If Applicable) or Substances	Major Pathologies	Onset	Adverse Event
West et Thorpe, 2011 [96]	1	23/F	Lucozade energy	N/A	Consumption of 2 L of “lucozade energy” per day in the last 3 months of pregnancy.	The infant was born with macrosomia because of refractory hyperinsulinism
Zekavat et al., 2017 [97]	1	23/F	ED	N/A	Ingestion of high-energy drinks with inadequate dietary intake in the past 6 months.	Severe menorrhagia because of vitamin K deficiency
Tinawi, 2022 [98]	1	40/M	ED	N/A	Consumption of 355 mL of ED after exercise	Severe rhabdomyolysis

**Table 7:** The results of our review on autoimmune and skin side effects.

References	N° Cases	Age/Sex	Energy Drink (If Applicable) or Substances	Major Pathologies	Onset	Adverse Event
Gerqari et al., 2016 [99]	1	19/M	ED	N/A	Every time, he drank the same taurine-containing energy drink.	Erythema exudative multiforme
Lee et al., 2013 [100]	1	33/F	ED	N/A	After taking ED containing 1000 mg of synthetic taurine.	Erythema, lips angioedema

## Data Availability

The data presented in this study are available in Table 2, Table 3, Table 4, Table 5, Table 6 and Table 7.
